# Distilled Water Dilution Increases Measured Fecal pH in Fresh Canine Feces

**DOI:** 10.3390/ani16182894

**Published:** 2026-09-14

**Authors:** Pedro Henrique Marchi, Carolina Dantas Micheletti, Jennifer Machado de Souza, Gabriela Luiza Fagundes Finardi, Eduarda Lorena Fernandes, Nicholas Sartini Santiago, Luana Dias dos Santos, Matheus Henrique dos Santos, Júlio Cesar de Carvalho Balieiro, Thiago Henrique Annibale Vendramini

**Affiliations:** Pet Nutrology Research Center, Department of Animal Nutrition and Production, School of Veterinary Medicine and Animal Science, University of Sao Paulo, Pirassununga 13635-900, SP, Brazil; pedro.henrique.marchi@usp.br (P.H.M.); carolinadantas@usp.br (C.D.M.); jennifersouza790@usp.br (J.M.d.S.); gabriela.finardi@usp.br (G.L.F.F.); eduarda_lorena@usp.br (E.L.F.); nicholas.santiago@usp.br (N.S.S.); luanads@usp.br (L.D.d.S.); math_henrique@usp.br (M.H.d.S.); balieiro@usp.br (J.C.d.C.B.)

**Keywords:** analytical standardization, potentiometry, sample preparation

## Abstract

Fecal pH is used in canine nutrition studies as an indirect indicator of intestinal fermentation, but sample preparation may affect the measured value. This study evaluated the effect of different feces-to-water dilution ratios on pH determination in canine feces. Thirty-seven samples from 20 healthy dogs were evaluated using five preparation protocols: direct measurement without dilution and dilution with distilled water at 1:1, 1:2, 1:5, and 1:10 feces-to-water ratios, on a weight-to-volume basis. Sample preparation affected fecal pH (*p* < 0.0001). The least-squares mean pH was 6.37 for direct measurement, 6.54 for 1:1, 6.71 for 1:2, 6.88 for 1:5, and 7.04 for 1:10. Fecal pH increased progressively with increasing water addition, with significant differences among all preparation protocols. Regression analysis confirmed that water addition increased measured fecal pH, and the response was better described by a quadratic model than by a linear model. These findings show that the amount of water added is a relevant methodological factor in fecal pH determination. Among the protocols evaluated, the 1:1 feces-to-water ratio appears to be the most suitable standardized method for routine analysis because it used the lowest amount of water while providing adequate conditions for homogenization and electrode contact.

## Simple Summary

Measuring the acidity level of feces can help researchers understand how diet affects the activity of bacteria in the intestine of dogs. However, there is no single standard way to prepare fecal samples before measurement. Many studies dilute feces with distilled water in different proportions to make the sample easier to handle, but this may change the value obtained and make comparisons among studies difficult. This study evaluated whether adding different amounts of distilled water changes fecal acidity measured in fresh dog feces. Samples from healthy dogs were measured directly, without dilution, and after dilution with increasing amounts of water. The results showed that adding water increased the measured value, and the increase became greater as more water was added. Because direct measurement may not always be feasible or analytically appropriate for a semi-solid fecal matrix, the 1:1 feces-to-water dilution may represent a practical method for fecal pH determination. The 1:1 ratio required the smallest amount of added water among the dilution protocols evaluated while providing suitable conditions for sample homogenization and measurement. These findings may help improve standardization in studies evaluating intestinal health and nutrition in dogs.

## 1. Introduction

Fecal pH is widely used in canine nutrition research as a practical and non-invasive indicator of colonic fermentation and microbial metabolic activity [1]. Although it does not directly quantify fermentation metabolites, fecal pH reflects the balance between saccharolytic and proteolytic processes occurring in the large intestine. Fermentation of non-digestible carbohydrates produces short-chain fatty acids (SCFAs), mainly acetate, propionate, and butyrate, which contribute to a more acidic intestinal environment [2,3,4,5,6]. In contrast, proteolytic fermentation favors the production of ammonia, phenols, indoles, and other alkaline metabolites that may increase fecal pH [7,8,9,10,11,12]. Therefore, fecal pH is frequently evaluated together with fecal SCFA concentrations, ammonia, score, and microbiota and nutrient digestibility to characterize intestinal responses to dietary interventions [1,2,7,8,9,10].

Diet composition directly influences the substrates available for microbial fermentation and is therefore one of the main determinants of fecal pH in dogs. Prebiotics, probiotics, and fermentable fibers may stimulate saccharolytic bacteria, increase SCFA production, and reduce the concentration of alkaline metabolites [1,7,9]. Conversely, diets containing greater amounts of protein reaching the colon may enhance proteolytic fermentation and modify the intestinal microbial ecosystem [8,10,12]. However, changes in microbial populations or fermentation end-products do not always result in detectable differences in fecal pH [13,14,15]. Because the biological effects investigated in nutritional studies may be relatively subtle, the reliability of fecal pH as a complementary outcome depends on the use of a consistent and reproducible analytical method.

Despite its frequent use, there is no standardized protocol for measuring fecal pH in dogs. Procedures vary regarding the interval between defecation and analysis, sample storage, homogenization, electrode type, equilibration time, and preparation of the fecal material [1,16,17,18,19]. Continued microbial activity and volatilization of fermentation metabolites after defecation may alter the physicochemical characteristics of the sample, while refrigeration and freezing may differently affect fermentation-related measurements [17,18,19,20]. These sources of methodological variation can reduce analytical reproducibility and complicate comparisons among studies, even when similar animals, diets, or nutritional interventions are evaluated.

A particularly important source of variation is the amount of distilled water added before pH determination. Fecal samples are commonly diluted to improve homogenization and electrode contact with the heterogeneous, semi-solid matrix [1]. However, feces-to-water ratios differ considerably among studies, with protocols such as 1:2, 1:5, and 1:10 being frequently reported [1,17]. Although dilution facilitates sample handling, the addition of water may modify the concentration of organic acids, ionic strength, buffering capacity, and electrochemical conditions of the sample, thereby altering the measured pH rather than merely assisting its determination [21,22,23,24,25]. Evidence from horses has shown that water addition can affect mean fecal pH and measurement variability [17], but the magnitude and direction of this effect have not been adequately characterized in canine feces.

Establishing how dilution influences fecal pH is essential for the interpretation of previous studies and for the development of a standardized protocol in canine nutrition research. Therefore, the present study evaluated fecal pH in fresh samples subjected to different feces-to-water ratios and compared these values with measurements obtained without water addition. We hypothesized that increasing the amount of distilled water would systematically alter the measured pH. By determining whether commonly used dilution protocols produce comparable results, this study aims to improve the reproducibility, interpretation, and comparability of fecal pH measurements in dogs.

## 2. Materials and Methods

### 2.1. Ethical Statement

This study was conducted in accordance with the Ethical Principles in Animal Research and was approved by the Ethics Committee on Animal Use of the School of Veterinary Medicine and Animal Science, University of Sao Paulo (CEUA/FMVZ-USP), under protocol number 7464060223.

### 2.2. Animals, Diet, and Housing

The experiment was conducted at the Pet Nutrology Research Center (CEPEN Pet), Department of Animal Nutrition and Production, School of Veterinary Medicine and Animal Science, University of São Paulo, Brazil. Twenty healthy adult dogs from the research facility were included, comprising twelve Beagles (body weight: 10.21 ± 1.21 kg; food intake: 170.92 ± 34.18 g/day; age: 2.70 ± 1.08), six Brazilian Terriers (body weight: 7.87 ± 1.60 kg; food intake: 159.00 ± 56.73 g/day; age: 1.46 ± 0.11), and two Labrador Retrievers (body weight: 23.90 ± 2.29 kg; food intake: 236.75 ± 36.13 g/day; age: 6.79 ± 0.07), of both sexes and with an ideal body condition score. Health status was confirmed by physical examination and nutritional history assessment before the beginning of the study.

Throughout the experimental period, all dogs received the same complete and balanced commercial dry diet. The diet met the nutritional recommendations established by FEDIAF for adult dogs [26]. The amount of food offered to each animal was based on its historical food intake and was adjusted according to body weight and body condition score [27,28], which were assessed every two weeks throughout the study.

The commercial dry diet used to standardize all dogs throughout the study was composed of poultry by-product meal, pork crackling meal, corn gluten meal, cassava meal, whole genetically modified corn, sorghum grain, broken rice, defatted rice bran, dried beet pulp, refined lard, chicken fat, refined fish oil, potassium chloride, sodium chloride, natural antioxidants, bentonite, *Yucca schidigera* extract, hydrolyzed poultry and porcine liver, yeast cell wall, vitamins, and mineral sources. Its guaranteed nutrient composition is presented in Table 1. All dogs had ad libitum access to fresh water.

The dogs were routinely housed in the same facility, in collective kennels with 3.42 m^2^ of covered area and 7.21 m^2^ of uncovered area, with concrete floors and tiled walls, and were generally housed in pairs. They were released into grass-covered play areas twice daily for exercise and social interaction. On the day before fecal collection, the animals were allocated to separate kennels to allow individual identification of the samples. During the collection period, the kennels were inspected every 15 min for spontaneous defecation, and feces were collected immediately after elimination to ensure that fresh samples were obtained. After fecal collection, the dogs were released into their usual grass-covered play areas.

### 2.3. Experimental Design, Fecal Sample Collection, and Preparation

This study was designed as a repeated-measures methodological evaluation in which each fecal sample served as its own control. All dogs had been receiving the same experimental diet at stable individual amounts for at least two months before sample collection, with food allowance adjusted as necessary to maintain body weight and ideal body condition score. Fecal collections were performed on two separate days, 20 days apart. On the first collection day, fresh fecal samples were obtained from all 20 dogs, whereas on the second collection day, samples were obtained from 17 dogs because fresh feces could not be collected from three animals under the established collection conditions. Therefore, a total of 37 fecal samples were obtained, with 17 dogs contributing two samples and three dogs contributing one sample.

Fresh fecal samples were collected immediately after spontaneous defecation, and samples contaminated with urine, soil, or other foreign material were excluded. Immediately after collection, the feces were manually homogenized to obtain a uniform fecal matrix. Each fecal sample was evaluated under five preparation conditions: direct measurement without dilution and sequential dilution with distilled water at feces-to-water ratios of 1:1, 1:2, 1:5, and 1:10.

Direct pH measurement was performed in the freshly homogenized fecal matrix. For the dilution conditions, a single 3-g aliquot from the same homogenized sample was weighed using an analytical balance with a precision of 0.001 g and progressively diluted throughout the procedure. Initially, 3 mL of distilled water was added to the 3-g fecal aliquot to obtain the 1:1 feces-to-water ratio. After homogenization and pH measurement, an additional 3 mL of distilled water was added to the same suspension to obtain the 1:2 ratio, corresponding to a total water volume of 6 mL. Subsequently, an additional 9 mL was added to obtain the 1:5 ratio, corresponding to a total water volume of 15 mL. Finally, an additional 15 mL was added to obtain the 1:10 ratio, corresponding to a total water volume of 30 mL.

After each addition of distilled water, the suspension was manually homogenized with a sterile spatula until a visually uniform consistency was obtained. The pH was measured before proceeding to the subsequent dilution step. The use of the same homogenized fecal sample for direct measurement and of the same 3-g aliquot for all sequential dilution conditions was adopted with the aim of minimizing biological variation among preparations and allowing the effect of increasing dilution on fecal pH determination to be evaluated. Distilled water was selected as the diluent because it is commonly used in studies evaluating fecal fermentation and gastrointestinal health in dogs.

To assess whether the time required for the sequential measurements could influence fecal pH, one fecal sample on each collection day was used as a temporal control. Four preparations corresponding to the 1:1, 1:2, 1:5, and 1:10 feces-to-water ratios were prepared from the same homogenized fecal sample, and pH was measured immediately after preparation and again after 15 and 30 min. These time points covered the maximum period required to complete the sequential analysis of an experimental sample.

### 2.4. pH Meter Calibration and Measurement Procedure

Fecal pH was measured using a digital potentiometer (Starter 3100C, OHAUS Corporation, Parsippany, NJ, USA) equipped with a penetration electrode suitable for semi-solid biological samples. Before each analytical session, the pH meter was calibrated at room temperature using certified buffer solutions at pH 4.00 and 7.00, according to the manufacturer’s recommendations. Calibration was considered acceptable when the electrode slope was between 95% and 105%.

For direct pH determination, the penetration electrode was inserted directly into the freshly homogenized fecal matrix without the addition of water. Care was taken to ensure adequate contact between the electrode sensing surface and the fecal material. For the dilution conditions, the fecal suspension was gently stirred immediately before each measurement to reduce sedimentation, and the electrode was immersed directly into the homogenized suspension. The measurements were performed sequentially in the same 3-g aliquot, following the fixed order of 1:1, 1:2, 1:5, and 1:10.

For both direct and diluted preparations, the pH value was recorded after the digital reading had stabilized. Between consecutive measurements, the electrode was rinsed with distilled water and gently dried with lint-free tissue to reduce sample carryover and measurement errors. All measurements were performed immediately after homogenization and under the same laboratory conditions to limit potential changes associated with ongoing microbial fermentation. During the analytical procedure, the temperature of the undiluted fecal samples, the temperature and pH of the distilled water, and the elapsed time from fecal collection to completion of the sequential pH measurements were recorded.

### 2.5. Statistical Analysis

Data were analyzed using linear mixed models. For the comparison among sample preparation protocols, preparation protocol was included as a fixed effect and fecal sample nested within dog was included as a random effect to account for the non-independence of observations obtained from the same sample. Fecal pH values obtained by direct measurement and by each dilution protocol were compared using least-squares means, followed by the Tukey–Kramer adjustment for multiple comparisons. Differences were considered significant at *p* < 0.05.

To evaluate the relationship between the amount of distilled water added and measured fecal pH, water addition was also analyzed as a quantitative variable using linear and quadratic mixed-effects regression models. Models were fitted using restricted maximum likelihood, with a variance-components covariance structure. Regression coefficients and their standard errors were estimated for both models. Model fit was evaluated using the Akaike information criterion (AIC), with lower values indicating better fit. Model residuals were evaluated to assess the assumptions of normality and homogeneity of variance.

## 3. Results

**Figure 1:**
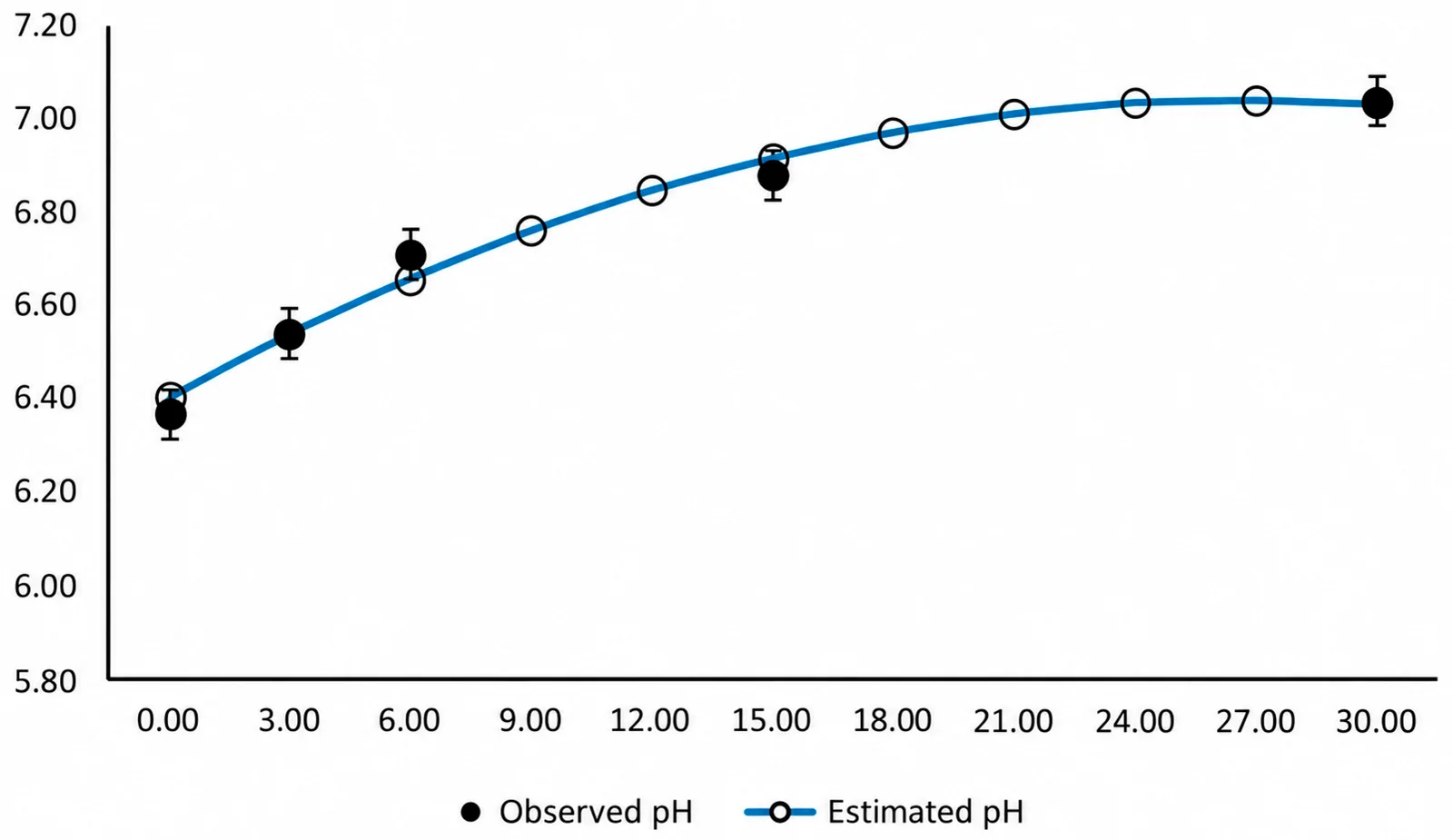
Quadratic relationship between the volume of distilled water added and measured fecal pH in fresh canine feces. Filled circles represent observed pH values, and open circles connected by the line represent estimated pH values from the quadratic regression model. Error bars represent the standard error of the mean.

A total of 37 fecal samples were obtained from 20 dogs submitted to five sample preparation protocols, resulting in 185 pH determinations included in the statistical analysis. Considering all measurements, fecal pH ranged from 5.83 to 7.66, with an overall mean of 6.71, a standard deviation of 0.39, and a coefficient of variation of 5.79%. Sample preparation affected fecal pH values (*p* < 0.0001). The least-squares mean pH was 6.37 for direct measurement, 6.54 for the 1:1 dilution, 6.71 for the 1:2 dilution, 6.88 for the 1:5 dilution, and 7.04 for the 1:10 dilution (Table 2; Figure 1).

Measurement without dilution produced lower pH values than all dilution conditions (*p* < 0.05). In addition, each successive dilution resulted in a higher pH than the preceding condition (*p* < 0.05). When compared with measurement without dilution, the mean fecal pH increased by 0.17 units with the 1:1 dilution, 0.34 units with the 1:2 dilution, 0.51 units with the 1:5 dilution, and 0.67 units with the 1:10 dilution. Overall, increasing amounts of added water resulted in progressively higher measured fecal pH.

The mean elapsed time from fecal collection to completion of the sequential pH measurements was 21.76 ± 4.93 min, ranging from 12 to 28 min. At the time of direct measurement, the undiluted fecal samples had a mean temperature of 26.45 ± 1.24 °C (range: 23.8–28.7 °C). The distilled water used for dilution was maintained at room temperature; its temperature was 22.0 °C on the first collection day and 22.2 °C on the second. The corresponding pH values of the distilled water were 6.25 and 6.26, respectively.

The temporal controls showed minimal variation in fecal pH during the 30-min period evaluated. Across both collection days and all dilution conditions, the difference between the initial measurement and the measurement obtained after 30 min ranged from 0.00 to 0.03 pH units (Table 3). Temporal controls also showed a progressive increase in pH with increasing dilution, although each dilution ratio was prepared independently rather than sequentially. At 0 min, pH increased from 6.44 at 1:1 to 7.02 at 1:10 on day 1 and from 6.75 to 7.27 on day 2. These changes over time were small compared with the differences observed among dilution levels in the main experiment.

Regression analysis confirmed the association between the amount of distilled water added and measured fecal pH. The linear mixed-effects model was significant (*p* < 0.0001), with an estimated increase of 0.02049 pH units per additional milliliter of distilled water. The quadratic mixed effects model also showed significant linear (*p* < 0.0001) and quadratic (*p* < 0.0001) terms. Regression coefficients, 95% confidence intervals, and model-fit statistics are presented in Table 4 and Table 5.

Based on the lower AIC, the quadratic model provided a better fit than the linear model. The fitted equation was:

pH measured = 6.4007 + 0.04799D − 0.0009D^2^

In this equation, D represents the volume of distilled water added (mL) to the 3-g fecal sample. The intercept (6.4007) represents the estimated fecal pH at 0 mL of added water. The positive linear coefficient (0.04799) describes the initial increase in measured pH with water addition, whereas the negative quadratic coefficient (−0.0009) indicates that the magnitude of this increase progressively decreases as the volume of added water increases.

The fitted quadratic curve showed a progressive increase in measured fecal pH, with the slope decreasing as the amount of added water increased (Figure 1). Nonetheless, the fitted equation is descriptive only of the experimental conditions evaluated in the present study, corresponding to a 3-g fecal sample and distilled water additions ranging from 0 to 30 mL. These findings demonstrate that water addition systematically affects fecal pH determination, with measured pH increasing progressively as the amount of distilled water added increased.

## 4. Discussion

The present study demonstrated that the addition of distilled water influences pH determination in fresh canine feces. Measured pH increased as the amount of water added to the sample increased, with significant differences among all preparation conditions. These findings indicate that dilution is not merely a sample preparation step but an analytical factor capable of influencing the resulting pH value. Consequently, differences in feces-to-water ratios among studies may limit direct comparisons, even when the same analytical endpoint is being evaluated [1,17,18].

Although fecal pH is an indirect marker, it is a practical screening tool for intestinal assessment in dogs because it is rapid, noninvasive, inexpensive, and requires minimal equipment. It may therefore provide useful complementary information when investigating dietary interventions capable of modifying colonic fermentation, including prebiotics, probiotics, fermentable fibers, and different protein sources [11,29]. Because the magnitude of diet-induced changes in fecal pH may be small, methodological variability introduced during sample preparation can become particularly relevant. Standardization is therefore essential if fecal pH is to be used reliably for treatment comparisons and screening purposes.

The increase in measured pH following dilution likely reflects changes in the physicochemical properties of the fecal matrix. Importantly, the pH of the distilled water (6.25 and 6.26) was lower than all fecal pH values measured, indicating that the increase observed with dilution did not result from a simple shift toward the pH of the diluent. The addition of water decreases the concentration of soluble fermentation products, including organic acids, and may alter ionic strength, buffering capacity, and hydrogen ion activity [1,18,21,25]. Because pH electrodes respond to hydrogen ion activity in the surrounding aqueous phase, these changes may directly influence the recorded value. At the same time, dilution facilitates sample homogenization and improves contact between the electrode and the sample. Thus, water addition has two analytical consequences, it facilitates measurement while simultaneously modifying the matrix being measured.

A similar effect has been described in equine feces, in which both measured pH and measurement variability were influenced by the amount of distilled water added during sample preparation [17]. Based on those findings, measurement of undiluted fecal liquid was recommended when a sufficient liquid fraction could be obtained [17]. Fecal dry matter in horses may vary considerably according to management conditions, with values ranging from 18.7% in horses maintained at pasture to 27.2% after transition to a stabled management system [30]. In dogs, fecal dry matter is also variable, and values around 30% have been reported in healthy animals [31]. However, canine feces typically have a semi-solid consistency that may hinder the recovery of sufficient free liquid for pH determination. Therefore, the undiluted fecal-liquid approach proposed for horses may be less practical in dogs, supporting the use of a standardized dilution procedure for routine analysis.

Direct measurement was included in the present study as a reference for evaluating the effect of water addition and to determine whether electrode insertion into fresh canine feces was technically feasible. Because it represents the least altered condition of the fecal sample, direct measurement was considered the most appropriate reference available for comparison with the dilution protocols. However, its routine use presents important limitations. Variations in fecal consistency and moisture may affect electrode contact and reading stabilization, and insertion of the electrode into a semi-solid matrix does not provide the same measurement conditions as a homogenized fecal suspension. Therefore, direct measurement was used as an experimental reference, despite these analytical limitations, because no better reference condition was available.

Different feces-to-water ratios have been used for pH determination in canine studies, with no consistently adopted preparation method [1,15,29]. In the present study, the 1:1 dilution required the smallest amount of added water while providing adequate homogenization and electrode contact. These characteristics make the 1:1 feces-to-water ratio the most suitable standardized protocol among those evaluated, as it improves analytical feasibility while limiting the alteration of the original fecal matrix caused by dilution.

The relationship between water addition and measured pH was better described by a quadratic than by a linear model, indicating that the effect of dilution was not proportional across the evaluated range. The fitted curve showed an initial increase in pH, with the slope decreasing at higher amounts of added water. This nonlinear response further emphasizes that results obtained using different dilution ratios cannot be readily converted or compared without considering the specific preparation method.

Beyond the feces-to-water ratio, other analytical conditions should also be standardized. Based on the present findings, canine fecal pH should preferably be determined in fresh, thoroughly homogenized samples prepared at a 1:1 feces-to-water ratio on a weight-to-volume basis. The pH meter should be calibrated before each analytical session, and the same electrode, equilibration time, reading stabilization criterion, and cleaning procedure should be applied consistently. Studies should also report the dilution ratio, interval between defecation and analysis, sample temperature, electrode type, and storage conditions to improve methodological reproducibility and comparisons across experiments.

Overall, the present findings support the use of a standardized 1:1 feces-to-water dilution for routine pH determination in canine feces. The protocol provides suitable conditions for homogenization and electrode contact while minimizing the analytical change associated with water addition. Given its simplicity, low cost, and applicability as a screening measure of the intestinal environment, fecal pH can provide useful complementary information in canine nutritional studies, provided that sample preparation and analytical conditions are carefully standardized.

One limitation of the present study is that the dilution conditions were evaluated sequentially in a fixed order using the same fecal aliquot, which does not completely separate dilution from other possible order-related effects. The use of independent aliquots could reduce this potential effect, but it could also introduce additional variation due to the heterogeneous nature of the fecal matrix, even after careful homogenization. Importantly, in the temporal controls, the four dilution ratios were prepared independently from the same homogenized fecal sample and still showed a progressive increase in pH with increasing dilution. This pattern was observed on both collection days, supporting that the increase found in the main experiment was not solely related to the sequential order of dilution. However, these controls were performed using only two fecal samples, and this finding should therefore be interpreted with caution.

Another limitation is that all dogs were healthy, maintained on the same diet, and housed at a single research facility. Therefore, the magnitude of the effect of dilution may differ in other populations or under different dietary and management conditions. In addition, fecal dry matter was not measured, and its potential influence on the response of the fecal matrix to dilution could not be directly assessed. Accordingly, the present findings should be interpreted within the experimental conditions evaluated in this study.

## 5. Conclusions

The addition of distilled water progressively increased the measured fecal pH. Among the tested dilution protocols, the 1:1 feces-to-water dilution required the lowest amount of added water while providing suitable conditions for homogenization and electrode contact. Therefore, among the conditions evaluated, the 1:1 dilution appears to be the most practical option for routine fecal pH determination. Although direct measurement served as a useful experimental reference, it is less suitable for routine analysis.

## Figures and Tables

**Table 1:** Ingredient and nutrient composition of the experimental diet fed to dogs throughout the study.

Nutrient	Guaranteed Level
Moisture (max.), %	10.00
Crude protein (min.), %	26.00
Ether extract (min.), %	15.00
Ash (max.), %	7.50
Crude fiber (max.), %	3.00
Calcium (min.), %	0.70
Calcium (max.), %	1.50
Phosphorus (min.), %	0.60
Sodium (min.), %	0.20
Potassium (min.), %	0.58
Omega-6 fatty acids (min.), %	2.50
Omega-3 fatty acids (min.), %	0.28
Beta-glucans (min.), %	0.06
Mannan-oligosaccharides (min.), %	0.03
Metabolizable energy, kcal/kg	4052 kcal/kg

Note: The diet contains genetically modified corn and corn gluten derived from events involving *Bacillus thuringiensis*, *Agrobacterium tumefaciens*, *Streptomyces viridochromogenes*, and/or *Zea mays*.

**Table 2:** Fecal pH values obtained by direct measurement and after different feces-to-water dilution ratios in fresh canine feces.

Variable	No Dilution	1:1	1:2	1:5	1:10	SEM	*p*
Mean pH	6.37 ^e^	6.54 ^d^	6.71 ^c^	6.88 ^b^	7.04 ^a^	0.05	<0.0001

Means followed by different lowercase letters differ significantly according to the Tukey–Kramer test (*p* < 0.05). SEM = standard error of the mean.

**Table 3:** Temporal stability of fecal pH measurements at different feces-to-water ratios during the maximum analytical period.

Collection Day	Feces-To-Water Ratio	0 min	15 min	30 min	ΔpH (30–0 min)
Day 1	1:1	6.44	6.46	6.47	+0.03
Day 1	1:2	6.62	6.63	6.63	+0.01
Day 1	1:5	6.79	6.79	6.80	+0.01
Day 1	1:10	7.02	7.01	7.02	0.00
Day 2	1:1	6.75	6.76	6.77	+0.02
Day 2	1:2	6.95	6.94	6.96	+0.01
Day 2	1:5	7.09	7.10	7.10	+0.01
Day 2	1:10	7.27	7.28	7.28	+0.01

ΔpH = pH at 30 min − pH at 0 min. On each collection day, the temporal control was performed using four preparations obtained from the same homogenized fecal sample.

**Table 4:** Parameter estimates for the linear and quadratic mixed-effects regression models describing the relationship between distilled water addition and measured fecal pH.

Model	Parameter	Estimate ± SE	95% CI	*F* (df)	*p*
Linear	Intercept	6.4878 ± 0.04995	6.3865–6.5891	—	<0.0001
	Water volume	0.02049 ± 0.000915	0.01868–0.02230	501.64 (1147)	<0.0001
Quadratic	Intercept	6.4007 ± 0.05028	6.2987–6.5027	—	<0.0001
	Water volume	0.04799 ± 0.002793	0.04247–0.05351	295.24 (1146)	<0.0001
	Water volume^2^	−0.000900 ± 0.0000879	−0.001074 to −0.000726	103.50 (1146)	<0.0001

Values are presented as parameter estimates ± standard error (SE), with corresponding 95% confidence intervals (CI), *F* statistics, degrees of freedom (df), and *p* values. Water volume represents the amount of distilled water added, in mL, to the 3-g fecal sample. In the quadratic model, water volume^2^ represents the quadratic term of the regression.

**Table 5:** Model-fit statistics for the linear and quadratic mixed-effects regression models describing the relationship between distilled water addition and measured fecal pH.

Model	AIC	AICc	BIC	ΔAIC
Linear	−79.9	−79.9	−76.7	62.0
Quadratic	−141.9	−141.8	−138.6	0

AIC = Akaike information criterion; AICc = corrected Akaike information criterion; BIC = Bayesian information criterion; ΔAIC = difference between the AIC of each model and the lowest AIC among the evaluated models. Lower values indicate better relative model fit.

## Data Availability

The data presented in this study are available on request from the corresponding author.

## Abbreviations

The following abbreviations are used in this manuscript:

AIC	Akaike information criterion
CEUA/FMVZ	Ethics Committee on Animal Use of the School of Veterinary Medicine and Animal Science
CEPEN Pet	Pet Nutrology Research Center
SCFA	Short-chain fatty acid
SEM	Standard error of the mean
